# Genetic effects on the efficiency and responsiveness to phosphorus use in popcorn as estimated by diallel analysis

**DOI:** 10.1371/journal.pone.0216980

**Published:** 2019-05-16

**Authors:** Ismael Fernando Schegoscheski Gerhardt, Antonio Teixeira do Amaral Junior, Guilherme Ferreira Pena, Lauro José Moreira Guimarães, Valter Jario de Lima, Marcelo Vivas, Pedro Henrique Araújo Diniz Santos, Fernando Rafael Alves Ferreira, Marta Simone Mendonça Freitas, Samuel Henrique Kamphorst

**Affiliations:** 1 Department of Plant Agriculture, University of Guelph, Guelph, ON, Canada; 2 Laboratório de Melhoramento Genético Vegetal, Universidade Estadual do Norte Fluminense Darcy Ribeiro, Campos dos Goytacazes, RJ, Brazil; 3 Universidade Estadual de Mato Grosso, Campus Alta Floresta, Alta Floresta, MT, Brazil; 4 Empresa Brasileira de Pesquisa Agropecuária, Embrapa Milho e Sorgo, Sete Lagoas, MG, Brazil; Louisiana State University College of Agriculture, UNITED STATES

## Abstract

Agricultural expansion and the need for sustainable cultivation are challenges faced by researchers involved in the generation of new cultivars that can adapt to abiotic stress. Knowledge of the genetic effects of characteristics related to efficiency and responsiveness to phosphorus use must be considered when implementing methods to obtain better genotypes. The aim of this study was to characterize and select popcorn hybrids based on their efficiency and responsiveness to phosphorus use, and estimate their combining abilities and genetic effects via diallel analysis to implement improvement programs for sustainable agriculture. Eight contrasting inbred lines were used to obtain simple hybrids for diallel analysis. Twenty-eight diallelic hybrids plus the popcorn parental lines were evaluated at two different sites under two contrasting environments for soil phosphorus availability (6 × 6 lattice design). Grain yield, popping expansion, and volume of expanded popcorn per hectare were measured. A combined analysis of variance and a test of means were performed. The classification and utilization of the phosphorus use efficiency index, according to the grain yield performance of the hybrids under contrasting environments, was considered. Through model 2 of the Griffing’s diallel analysis method, the general and specific combining abilities were estimated, along with their environmental interactions. The best strategy to obtain genotypes that are efficient and responsive to phosphorus involves exploring popcorn hybrids using genitors that result in the accumulation of additive genes that promote popping expansion. Hybrids P7 × L80, P7 × L59, P7 × L76, and P6 × L80 presented promising results and may be evaluated as cultivation options in phosphorus-deficient soils.

## Introduction

With the climate changes observed in the past few years, the promotion of more sustainable agriculture that is adapted to different abiotic stresses has become imperative [1]. One challenge is to obtain more efficient cultivars that are adapted for low-fertility soils to mitigate the use of agricultural inputs, and consequently reducing environmental pollution and production costs [2].

Phosphorus is one of the most important primary macronutrients for most cultivated crops, due to its participation in vital plant functions [3,4]. To fulfill the increasing demand for fertilizers, mining operations increased phosphate rock extraction five-fold between the 1960s and 2011 [5], and the global production of phosphate fertilizers has increased at a rate of 4% per annum [6]. As this is a non-renewable natural resource, with reserves that will be depleted within the next 50 years [7], improving plants to promote efficiency and responsiveness to phosphorus use is important. In addition, improved phosphorus efficiency and responsiveness are of interest for the sustainability of production systems, especially in tropical regions with low fertility soils, high acidity, and a high phosphorus adsorption capacity [8].

Knowledge of the genetic control of phosphorus-use efficiency and the predominant genes that express this characteristic is fundamental to adequately select improvement methods to be implemented to obtain better genotypes [9]. Studies in numerous crops have been undertaken to understand genetic control, identifying genomic regions, quantitative trait loci (QTL) mapping, and genes that may promote phosphorus-use efficiency, a task that has shown to be difficult due to its polygenetic inheritance and the strong influence by the environment [9–15]. However, there is no published literature regarding the genetic control of phosphorus-use efficiency in popcorn crops under field conditions.

In such a context, diallel analysis has been utilized in inheritance and genetic control studies related to important characteristics in many different crops. This methodology employs estimates of genetic parameters related to character control, as deduced from the additive and non-additive effects, to help select a more efficient selection method and to indicate the best genitors for hybridization that provide good genetic complementation for characteristics of economic interest [16–18]. Diallel analysis between inbred lines with contrasting efficiencies and responsiveness to phosphorus use may help select efficient genitors in low-fertility areas that are responsive to phosphorus application for the development of hybrids, and to indicate heterotic hybrids with satisfactory productivity in low-fertility areas.

Therefore, the purpose of this study was to evaluate the efficiency and responsiveness to phosphorus use in a set of popcorn hybrids and to estimate the general and specific combining abilities of their parental inbred lines to deduce their additive and non-additive genetic effects in a diallel analysis conducted in contrasting environments of soil nutrient availability.

## Material and methods

### Experimental conditions and hybrids

In a previous study, 25 S_7_ popcorn inbred lines from the UENF Breeding Program were classified and selected based on their efficiency and responsiveness to phosphorus use [19]. Of those, eight were selected for the present analysis, based on their allelic symmetry, as follows: three lines classified as efficient and responsive to phosphorus use (ER), three lines classified as inefficient and non-responsive to phosphorus use (INR), and two lines classified as intermediate in terms of efficiency and responsiveness to phosphorus use (Table 1).

**Table 1:** Popcorn lines utilized in the diallel analysis with their respective genealogies and classification regarding their efficiency and responsiveness to phosphorus use.

Lines	Origin population	PUE classification
P2	CMS-42 Composite: EMBRAPA	ER
P7	Zaeli hybrid	ER
L59	Beija-flor: UFV	ER
P6	Zaeli hybrid	Intermediate
L76	Viçosa: UFV	Intermediate
L77	Viçosa: UFV	INR
L75	Viçosa: UFV	INR
L80	Viçosa: UFV	INR

ER: efficient and responsive to phosphorus use; INR: inefficient and non-responsive to phosphorus use.

To obtain hybrids, controlled crossings followed the entire diallel scheme, comprising 28 simple hybrids. The eight genitors were inserted, and reciprocal hybrids were disregarded, for a total of 36 treatments.

The evaluation tests were conducted in the summers of 2015 and 2016 in two different sites and, in each site, with two contrasting environments in terms of soil phosphorus availability. The sites were Antônio Sarlo State Agricultural School, in the municipality of Campos dos Goytacazes–RJ, and the Experimental Station of the municipality of Itaocara–RJ.

The utilized experimental design was a 6 × 6 triple lattice. Sowing was performed according to the conventional planting system. Each experimental plot was comprised of a 5 m line, with a 0.70 m distance between the planting lines and 0.20 m between the plants, for a total of 25 plants per plot.

### Characterization of evaluated environments regarding their phosphorus availability

The experimental areas were pre-selected using soil chemical analysis in Campos dos Goytacazes and Itaocara to characterize those environments regarding their phosphorus availability, based on the samples collected from 0–10 and 10–20 cm layers, forming a sample composed of 10 subsamples (Table 2). The classification of phosphorus availability in the soil is based on the level of phosphorus and the clay content [20]. As the amount of clay in the soil increases, sorption capacity increases as well. Clay particles have a large amount of surface area where phosphate sorption can take place. The classification of phosphorus (P) availability in those two sites was low according to the clay content of their soils, which is characteristic of weathered tropical soils.

**Table 2:** Chemical and clay analysis of soil in the sites located in Campos dos Goytacazes and Itaocara, RJ.

Parameters measured	Campos dos Goytacazes	Itaocara
pH	6.0	5.8
P (mg/dm^3^)	7.0	10.0
K (mmol c/dm^3^)	4.3	3.2
Ca (mmol c/dm^3^)	17.9	24.9
Mg (mmol c/dm^3^)	13.5	29.8
Al (mmol c/dm^3^)	0	0
Na (mmol c/dm^3^)	1.3	1.4
C (g/dm^3^)	9.7	10.6
OM (g/dm^3^)	22.0	18.1
CEC (mmol c/dm^3^)	61.2	82.1
BS (%)	64.0	79.0
Clay (g/dm^3^)	305.0	140.0

pH: potential hydrogen; P: phosphorus; K: potassium; Ca: calcium; Mg: magnesium; Al: aluminium; Na: sodium; C: carbon; OM: organic matter; CEC: cation exchange capacity; BS: base saturation.

To simulate contrasting environments—either high or low phosphorus conditions—two different fertilization rates were used in order to stimulate the genotypes to express genes for phosphorous use efficiency and responsiveness. The environment with high-phosphorus availability included 30 kg ha^-1^ of N, 85 kg ha^-1^ of P_2_O_5_, and 40 kg ha^-1^ of K_2_O. The environment with low-phosphorus availability included 30 kg ha^-1^ of N, 0 kg ha^-1^ of P_2_O_5_, and 40 kg ha^-1^ of K_2_O. Topdressing was applied in both environments when the plants reached the V6 phenological stage at a concentration of 100 kg ha^-1^ of N.

The primary macronutrient supply capacity in the experimental areas was determined according to the recommended fertilization for popcorn. This considered the quantity of nutrients in the soil in the 0–20 cm layer, established by chemical analysis, except for phosphorus in the environment with low phosphorus availability, which was zero.

### Phenotyping

The following characteristics were evaluated: i) grain yield (GY)–all 25 plants per plot were harvested and threshed manually. The GY was expressed as the average grain production in the experimental unit in grams per plot, corrected to 13% humidity and extrapolated to kg ha^-1^; ii) popping expansion (PE)–determined in plastic containers without oil, with two replications per plot, using grain aliquots weighing 30 grams each. Popping was performed in a microwave oven 1200 Watts for 2 min, the expanded popcorn volume was measured in a 2,000 mL beaker, the resulting value was divided by the initial grain weight of 30 g, and the final result was expressed as mL g^-1^; and iii) the volume of expanded popcorn per hectare (PV)–obtained through the product between grain yield and popping expansion, divided by 1,000, and expressed as m^3^ ha^-1^.

### Classification methodology regarding efficiency and responsiveness to phosphorus use

The method used to identify and classify diallelic hybrids in terms of efficiency and responsiveness to phosphorus use was based on deviations from the average grain yield of each hybrid in relation to the average grain productivity of each environment. Averages were plotted on a scatterplot; the x-axis accounted for the high phosphorus level deviations (responsiveness to phosphorus use), and the y-axis accounted for low phosphorus level deviations (phosphorus-use efficiency).

The expression used to classify the hybrids in terms of their phosphorus-use efficiency was:

$$E_{use}=Y_{il}-Y_{el},$$

In which:

*Y*_*il*_: the average grain productivity of hybrid ‘i’ in the environment with low phosphorus availability; and

*Y*_*el*_: the average grain productivity in the environment with low phosphorus availability.

To classify the hybrids based on their responsiveness to phosphorus use, the following was considered:

$$R_{use}=Y_{ih}-Y_{eh}$$

In which:

*Y*_*ih*_: the average grain productivity of hybrid ‘i’ in the environment with high phosphorus availability; and

*Y*_*eh*_: the average grain productivity in the environment with high phosphorus availability.

Thus, the hybrids were distributed along four quadrants in the scatterplot, depending on their grain yield performance in the contrasting environments with regard to the phosphorus availability: efficient and responsive (ER); efficient and non-responsive (ENR); inefficient and responsive (IR); or inefficient and non-responsive (INR).

### Genetic and statistical analyses

The Genes software package [21] was utilized for genetic and statistical analyses. Based on the characteristics assessed in the treatments, an individual analysis of variance was first performed for the high- and low-phosphorus environments, followed by a combined analysis for both environments. Averages were clustered according to the Scott-Knott algorithm [22] with a 5% probability.

A diallel analysis determined the combining ability according to Method 2 of the Griffing’s Model [23], in which the **p(p+1)/2** combinations corresponding to the genitors and their F_1_ hybrids were evaluated, by employing Model B, considering a fixed effect for the genotypes. The effects of treatments on the general combining ability (GCA) and the specific combining ability (SCA) were dissected, considering the following statistical model:

$$Y_{ijk}=m+g_{i}+g_{j}+s_{ij}+l_{k}+{gl}_{ijk}+{sl}_{ijk}+e_{ijk},$$

In which:

*Y*_*ijk*_: the average value of the hybrid combination (*i* ≠ *j*) or the genitor (*i* = *j*) at the k-th location;

*m*: the overall average;

*g*_*i*_, *g*_*j*_: the effects of the general combining ability of the *i*-th and *j*-th genitors, respectively;

*s*_*ij*_: the effect of the specific combining ability for the crossings between the genitors of order *i* and *j*;

*l*_*k*_: the fixed effect of the k-th location;

*gl*_*ijk*_: the effect of the interaction of the general combining ability of the i-th and j-th genitors with the location;

*sl*_*ijk*_: the effect of the interaction of the specific combining ability for the crossings between the genitors of order *i* and *j* with the location; and

*e*_*ijk*_: the average experimental error.

The quadratic component estimators of the fixed effects associated with GCA (Φg) and SCA (Φs) were:

$$\Phi g=\frac{MSGxE-MSR}{p+2}$$

Φs=MSSxE−MSR

In which:

*p*: the number of genitors;

*MSGxE*: the mean square of the interaction between GCA and the environment;

*MSSxE*: the mean square of the interaction between SCA and the environment; and

*MSR*: the mean square of the residue.

## Results and discussion

### Efficiency and responsiveness to phosphorus use among popcorn hybrids

The overall average of all characteristics was lower in the low-phosphorus environment than in the high-phosphorus environment (Table 3). Low phosphorus levels accounted for the low averages observed for characteristics evaluated in environments with lower levels of nutrients in the soil. This is because phosphorus is a vital component of cells and has important roles in plant metabolism; it is involved in initial growth and development and is also responsible for growth, maturity, and seed formation [24].

**Table 3:** Estimates of the average grain yield (GY), popping expansion (PE), and volume of popcorn per hectare (PV) for the 36 popcorn genotypes cultivated in environments with low- and high-phosphorus availability.

Genotype	GY (Kg.ha^-1^)		PE (mL.g^-1^)		PV (m^3^.ha^-1^)	
	Low P	High P	Low P	High P	Low P	High P
**P2**	1437.35 e	1796.83 f	20.96 d	21.80 d	29.98 e	39.45 f
**P7**	1707.80 d	2127.77 e	27.22 b	30.80 a	46.13 d	65.07 d
**L59**	1905.77 d	2472.76 d	23.04 c	24.00 c	44.19 d	59.32 d
**P6**	1552.54 e	1729.02 f	24.01 c	26.70 c	37.69 e	46.47 f
**L76**	953.71 f	1124.97 g	26.29 b	27.90 b	25.64 f	32.16 g
**L77**	507.03 g	774.90 h	26.05 b	25.30 c	13.20 g	19.43 h
**L75**	386.35 g	504.50 i	20.00 d	20.10 d	7.67 g	9.95 h
**L80**	343.90 g	401.19 i	29.49 a	31.00 a	10.55 g	12.17 h
**P2 × P7**	2359.40 c	2006.47 e	25.22 b	27.48 b	58.99 c	55.37 e
**P2 × L59**	1951.18 d	1773.51 f	22.83 c	23.06 d	43.93 d	41.23 f
**P2 × P6**	1990.21 d	2184.32 e	22.72 c	25.00 c	44.52 d	54.57 e
**P2 × L76**	1814.20 d	2083.60 e	24.70 c	24.85 c	45.26 d	52.53 e
**P2 × L77**	2007.60 d	2159.76 e	23.63 c	25.73 c	46.79 d	56.05 e
**P2 × L75**	2491.73 c	2857.63 c	23.53 c	21.52 d	58.44 c	61.39 d
**P2 × L80**	1905.39 d	2134.87 e	24.49 c	26.58 c	46.62 d	56.68 e
**P7 × L59**	2905.84 b	3333.46 b	29.69 a	27.92 b	85.90 a	93.00 a
**P7 × P6**	2293.87 c	2613.54 d	28.33 a	30.56 a	65.68 b	80.63 c
**P7 × L76**	2960.35 b	3213.04 b	29.59 a	29.71 a	88.13 a	95.32 a
**P7 × L77**	2736.51 b	3106.86 b	27.36 b	30.58 a	75.07 b	95.05 a
**P7 × L75**	3058.55 b	3284.67 b	27.33 b	26.37 c	83.17 a	86.66 b
**P7 × L80**	3057.82 b	3134.64 b	29.32 a	31.44 a	88.72 a	98.12 a
**L59 × P6**	2690.12 b	2899.81 c	25.92 b	26.15 c	69.41 b	75.68 c
**L59 × L76**	1737.63 d	2061.84 e	23.91 c	25.29 c	41.30 d	51.75 e
**L59 × L77**	1750.21 d	2150.25 e	27.94 a	26.17 c	49.04 d	56.17 e
**L59 × L75**	2634.36 c	2952.55 c	20.67 d	21.96 d	54.25 d	64.53 d
**L59 × L80**	1555.62 e	2081.99 e	26.90 b	27.02 b	41.89 d	55.64 e
**P6 × L76**	2615.98 c	2878.44 c	25.81 b	27.94 b	67.38 b	80.71 c
**P6 × L77**	2754.53 b	3006.07 c	27.61 a	28.81 b	75.94 b	86.69 b
**P6 × L75**	3862.42 a	3994.75 a	23.26 c	24.60 c	90.19 a	98.66 a
**P6 × L80**	2810.30 b	3113.48 b	29.33 a	29.67 a	82.90 a	92.03 a
**L76 × L77**	1203.27 e	1503.63 f	25.68 b	27.96 b	31.04 e	42.02 f
**L76 × L75**	2257.84 c	2555.88 d	25.68 b	23.75 c	57.78 c	60.35 d
**L76 × L80**	1206.70 e	1295.69 g	27.72 a	28.81 b	32.90 e	37.09 g
**L77 × L75**	2765.39 b	3103.30 b	26.53 b	24.62 c	73.36 b	77.00 c
**L77 × L80**	1792.40 d	2006.10 e	28.49 a	29.69 a	50.85 d	59.89 d
**L75 × L80**	2605.31 c	2933.74 c	26.31 b	25.48 c	68.41 b	74.82 c
**Average**	2071.37	2315.44	25.77	26.56	53.69	61.77

Averages followed by the same letter in a column belong to the same group according to the Scott-Knott algorithm, with a 5% probability level.

**Fig 1:**
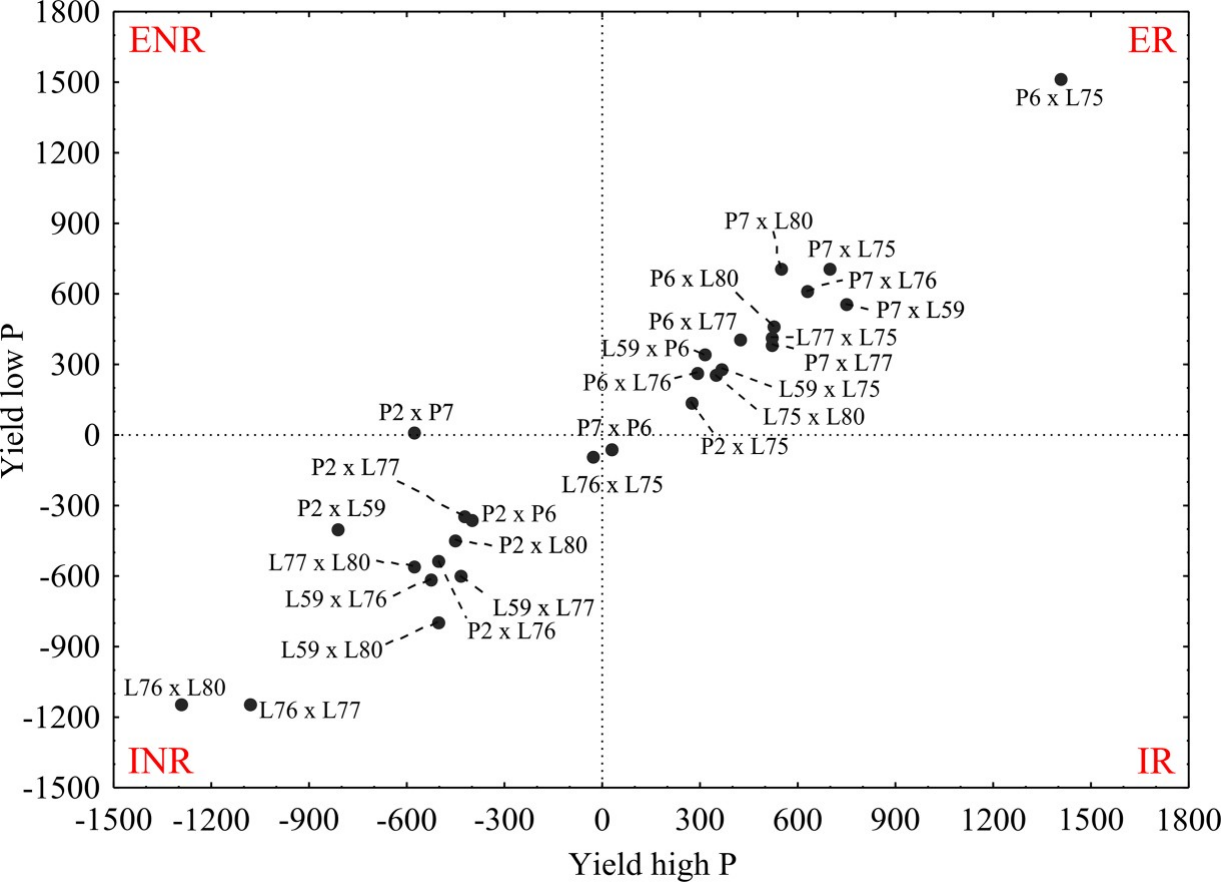
Graphical dispersion of 28 popcorn hybrids based on their phosphorus-use efficiency. ER = efficient and responsive; ENR = efficient and non-responsive; IR = inefficient and responsive; and INR = inefficient and non-responsive.

The graphical dispersion of hybrids in quadrants in terms of their efficiency and responsiveness to phosphorus use revealed that the P6 × L75, P7 × L75, P7 × L80, P7 × L76, P7 × L59, P6 × L80, P7 × L77, L77 × L75, P6 × L77, L59 × P6, L59 × L75, L75 × L80, P6 × L76, and P2 × L75 combinations were efficient and responsive (ER); that is, those genotypes expressed an average productivity that was superior to the averages found under the low- and high-phosphorus environments (Fig 1). Out of the 14 hybrids classified as ER, eight contained in their genealogy at least one genitor classified as efficient and responsive *per se*, and the rest were hybrids derived from INR or intermediary lines. The genitors from the most productive hybrid (P6 × L75, which produced 3,862.42 and 3,994.75 kg ha^-1^ in the low- and high-phosphorus environments, respectively), were previously classified as intermediary and INR, demonstrating that a crossing between genitors with the best production performances will not always provide the best hybrids. Thus, there is a need to reflect the importance of the allelic complementation effect due to the presence of dominance, especially when the purpose of the improvement program is to explore heterotic hybrids [25].

In contrast, hybrids L76 × L75, P2 × P6, P2 × L77, P2 × L80, P2 × L59, P2 × L76, L77 × L80, L59 × L77, L59 × L76, L59 × L80, L76 × L77, and L76 × L80 were classified as INR (Fig 1); that is, their average productivity is lower in environments with and without nutritional stress. Out of the 12 hybrids classified as INR, eight contained at least one genitor classified as inefficient and unresponsive in their genealogy. Genitors from the hybrid with the highest negative distinction (L76 × L80), based on the lowest average productivity estimate, were also classified as intermediary and INR.

### The combining ability of popcorn inbred lines for their efficiency and responsiveness to phosphorus use

There was a significant difference between the genotypes with a 1% probability for all the characteristics evaluated in the environments with low- and high-phosphorus levels in the soil, which indicates the presence of genetic variability among the evaluated genotypes (Table 4). Those genotypes may be utilized as a base population in improvement programs to ensure higher efficiency and responsiveness to phosphorus use.

**Table 4:** Combined Griffing’s diallel analysis for GY, PE, and PV of popcorn lines and hybrids evaluated under low- and high-phosphorus availability.

SV	DF	Mean squares					
		GY		PE		PV	
		Low P	High P	Low P	High P	Low P	High P
**Gen (G)**	35	3,897,128.83 **	4,180,396.59 **	40.74 **	51.04 **	3033.20 **	3387.81 **
**GCA**	7	5,047,841.13 **	5,356,317.21 **	150.97 **	243.75 **	4151.12 **	5438.51 **
**SCA**	28	3,609,450.75 **	3,886,416.43 **	13.19 ^ns^	2.86 ^ns^	2753.71 **	2875.13 **
**Environ (E)**	1	4,096,940.66 **	2,752,883.63 **	10.40 ^ns^	10.93 *	2138.33 **	3184.96 **
**G** × **E**	35	73,196.13 ^ns^	104,633.67 **	9.20 *	11.46 **	73.76 ^ns^	160.89 **
**GCA** × **E**	7	55,859.80 ^ns^	72,497.29 ^ns^	8.08 ^ns^	6.15 *	86.93 ^ns^	124.55 *
**SCA** × **E**	28	77,530.21 ^ns^	112,667.76 **	9.48 *	12.79 **	70.47 ^ns^	169.97 **
**Residual**	110	97,233.69	51494.42	6.37	3.01	97.63	52.60
**Average**		2071.37	2315.44	25.77	26.56	53.69	61.77
**Quadratic components of the fixed effects**				** **	** **	** **	** **
**GCA (Φg)**		80,889.56	87,555.47	2.30	3.96	65.93	88.89
**SCA (Φs)**		569,163.90	630,571.26	0.07	-0.53	426.41	461.66

^ns^: not significant

* and **: significant at 5 and 1% probability, respectively, according to the F test. Gen: genotypes; GCA: general combining ability; SCA: specific combining ability; Environ: environments (site). GY: grain yield; PE: popping expansion; and PV: volume of expanded popcorn per hectare.

The breakdown of genotypes in terms of their combining abilities revealed a significant effect of general combining ability (GCA) for the GY, PE, and PV characteristics under the low- and high-phosphorus environments (Table 4). This finding indicates the presence of additive effects in the expression of those characteristics, considering that at least one of the genitors differs from the others in relation to the quantity of favorable alleles with additive effects for such characteristics.

A significant effect was also observed in the specific combining ability (SCA) for the GY and PV characteristics in the low- and high-phosphorus environments (Table 4). This finding indicates that the non-additive genetic effects also influence the expression of those characteristics and highlight the existence of an allelic complementation effect between the genitors in the loci with some degree of dominance. The absence of significant SCA for PE reveals the predominance of the genetic additive effects to control that characteristic, corroborating previous findings [26–28].

Despite significance in the mean squares estimates, the quadratic components of the fixed effects (Φg e Φs) are those that most accurately reveal the predominance of the genetic effects of the evaluated characteristics [18]. For GY and PV, there was a predominance of the Φs estimate over Φg in the low- and high-phosphorus environments, indicating that non-additive effects are more important for the expression of such characteristics (Table 4). These results indicate that the best strategy to obtain genotypes that are efficient and responsive to phosphorus use for those characteristics should explore heterosis through the development and use of hybrids.

Non-additive genetic effects usually prevail for grain yield in popcorn, as previously reported [26,27,29]. Conversely, there is a contradiction for phosphorus-use efficiency, since researchers have highlighted the importance of both additive and non-additive effects in different crops. For example, [30] a study on the inheritance efficiency of many mineral elements found in soy genotypes determined that additive effects were more important for all elements, except for copper, manganese, and zinc; [9], a study reported the prevalence of non-additive effects in the genetic control of phosphorus-use efficiency in corn; [31] another study reported the predominance of genetic additive effects for nitrogen-use efficiency in wheat; [15] a study evaluating the genetic effects of characteristics related to phosphorus-use efficiency in soy crops reported a prevalence of non-additive effects; and [32] a further study reported that the actions of non-additive genes were more important for corn grain yield in terms of their tolerance to acid soils.

Selection of the diallel parental lines may interfere with the predominant expression of the genetic effects responsible for phosphorus-use efficiency. This is because both inbred lines, with a narrow genetic base, and varieties, with a broad genetic base [17], may be utilized. Therefore, each genitor may contribute differently, depending on the frequency of favorable alleles, thus providing contrasting results in relation to the predominant gene activity for a certain characteristic. The existing degree of genetic distance between parental lines also interferes with the genetic effects, since together with dominance deviations, it is utilized to estimate heterosis when identifying superior hybrids for phosphorus-use efficiency [33].

For PE, the Φg estimate was higher than the Φs estimate in environments with low and high soil phosphorus levels (Table 4). Thus, genetic additive effects are important for the expression of that characteristic, corroborating previous results from studies investigating the inheritance of expansion capacity in popcorn [34,26,35,36]. Such results indicate that adequate improvement strategies include intra-populational recurrent selection methods, to increase the frequency of alleles favorable to popping expansion.

Considering that efficiency and responsiveness to phosphorus use in popcorn are directly related to grain yield, in which non-additive genetic effects are greater than additive effects, the selection of lines to obtain superior hybrids must be carefully undertaken based on their genealogical contexts. Thus, the selection of genitors with high PE values–popcorn’s main qualitative characteristic—is an interesting strategy to aggregate superior qualities in hybrids according to the higher averages derived from additivity, enabling increased expression in both characteristics.

According to Sprague and Tatum [37], general combining ability refers to the average performance of a line in hybrid combinations and is expressed by the “ĝ_i_” estimate. When high positive or negative values are observed, the result is an “i” genitor that is superior or inferior to the remaining genitors of the diallel in relation to the average of its hybrids.

Regarding GY, the most outstanding lines with the best overall performances in the hybrid combinations due to their ĝ_i_ positive average magnitudes were: P7, P6, L75, and L59, in environments with low and high soil phosphorus levels (Table 5). This finding indicates that the stressful environment did not impose a behavioral differentiation in the lines when combined with the remaining components of the diallel, as observed through the non-significance of the GCA × E interaction for GY (Table 4).

**Table 5:** The average effect of the general combining ability (*ĝ*_*i*_) on grain yield, popping expansion, and popcorn volume in the inbred lines evaluated under low- and high-phosphorus environments.

Genitors	General combining ability (*ĝ*_*i*_)					
	GY		PE		PV	
	Low P	High P	Low P	High P	Low P	High P
**P2**	-124.79	-204.52	-2.28	-2.13	-7.87	-9.92
**P7**	414.56	410.93	1.94	2.66	15.47	17.84
**L59**	39.42	136.00	-0.80	-1.35	-0.91	0.07
**P6**	348.02	330.95	-0.09	0.71	8.82	10.60
**L76**	-293.89	-299.69	0.38	0.50	-6.81	-7.18
**L77**	-261.83	-225.32	0.74	0.51	-5.48	-4.42
**L75**	180.60	185.26	-1.86	-3.06	1.77	-1.26
**L80**	-302.1	-333.61	1.96	2.16	-4.98	-5.73

GY: grain yield; PE: popping expansion; and PV: volume of popcorn per hectare.

However, when classifying the lines in terms of phosphorus use, P7 and L59 were designated as efficient and responsive; P6 ranked as intermediate, and L75 was inefficient and non-responsive to phosphorus use (Table 1). Conversely, among the lines that presented a less significant ĝ_i_ values, L80, L76, L77, and P2, lines L80 and L77 were classified as inefficient and non-responsive; L76, as intermediate; and P2 as efficient and responsive to phosphorus use. These results suggest there is some agreement between the lines evaluated and those resulting from the combining ability estimates. Thus, in studies involving phosphorus stress, the value of the lines and their ĝ_i_ and ŝ_ij_ estimates should be considered, since the selection of a genotype does not indicate its general and specific combining abilities.

For the PE characteristic, lines L80 and P7 held the highest positive ĝ_i_ values in the environments with high and low phosphorus levels (Table 5), indicating that the use of those lines as genitors should increase hybrid averages. Conversely, the most outstanding negative lines for ĝ_i_, P2, L75, and L59, in the high- and low-phosphorus environments must be discarded in the crossings to obtain hybrids with high PE values. By providing positive ĝ_i_ values for GY and PE, P7 is characterized as a line of interest for crossings to obtain superior hybrids in terms of efficiency and responsiveness to phosphorus use.

Since PV positively and concomitantly associates grain yield and popping expansion, it led to an agreement in the higher and lower ĝ_i_ magnitude lines between GY and PE. Considering lines with the highest positive ĝ_i_ values for PV, P7, and P6, in the high- and low-phosphorus environments, P7 also comprised the groups with high ĝ_is_ values for PE and GY; and P6 belonged to the group with higher ĝ_i_ values for GY (Table 5). Among the lines with unfavorable ĝ_is_ values for PV (P2, L76, L77, and L80) in environments with high and low phosphorus levels, all were equally unfavorable for GY in both environments, and P2 was unfavorable for PE.

Analyzing the genealogical context in relation to the expressed results for general combining abilities regarding GY and PV, we may conclude that the lines derived from the “Zaeli” commercial hybrid were those that presented the highest ĝ_i_ values. This finding demonstrates that the genealogy has favorable genes and is of interest for both GY and for PV, whose characteristics are directly related to phosphorus use efficiency and responsiveness (Table 1). In relation to PE, the genealogies with favorable genes highlighted the “Zaeli” commercial hybrid and the “Viçosa: UFV” population, with the exception of line L75, which expressed negative ĝ_i_ values.

The specific combining ability is the behavior of a hybrid combination in relation to the average values of its genitors, expressed by the “ŝ_ij_” estimate. SCA occurs due to dominant genetic variance and other types of epistatic variance components. A promising hybrid must present a high estimate for specific combining ability, and at least one of its genitors must present high general combining ability [38].

For the GY characteristic, there was a predominance of dominant genetic effects for efficiency and responsiveness to phosphorus use. The six hybrids with the highest ŝ_ij_ estimates were: P6 × L75, P7 × L80, L77 × L75, P6 × L80, P7 × L76, and L75 × L80 (Table 6). In those combinations, at least one of the genitors expressed a high ĝ_i_ estimate, consistent with the expected result based on the general combining ability reported by Cruz et al. [38]. Notably, those hybrid combinations expressed high averages for GY, varying from 2,605.31 to 3,994.75 Kg ha^-1^ in environments with contrasting phosphorus use, and were included in the first and second groups of the Scott-Knott algorithm [22], with the exception of hybrid L75 × L80.

**Table 6:** Effect of specific combining ability (*ŝ*_*ij*_) on grain yield, popping expansion, and popcorn volume of the diallelic hybrids evaluated in low- and high-phosphorus environments.

Hybrids	Specific combining ability (*ŝ*_*ij*_)					
	GY		PE		PV	
	Low P	High P	Low P	High P	Low P	High P
**P2 × P7**	-1.74	-515.38	-0.20	0.38	-2.30	-14.32
**P2 × L59**	-34.82	-473.41	0.14	-0.03	-0.98	-10.69
**P2 × P6**	-304.39	-257.56	-0.67	-0.14	-10.11	-7.88
**P2 × L76**	161.51	272.36	0.84	-0.09	6.25	7.86
**P2 × L77**	322.86	274.15	-0.60	0.78	6.44	8.62
**P2 × L75**	364.55	561.45	1.91	0.14	10.85	10.80
**P2 × L80**	260.91	357.56	-0.96	-0.02	5.78	10.57
**P7 × L59**	380.49	471.10	2.78	0.05	17.65	13.32
**P7 × P6**	-540.08	-443.77	0.71	0.63	-12.30	-9.57
**P7 × L76**	768.31	786.36	1.51	-0.02	25.79	22.90
**P7 × L77**	512.41	605.81	-1.09	0.85	11.38	19.86
**P7 × L75**	392.02	373.05	1.49	0.21	12.24	8.32
**P7 × L80**	873.99	741.89	-0.35	0.05	24.54	24.25
**L59 × P6**	231.31	117.43	1.04	0.23	7.82	3.24
**L59 × L76**	-79.26	-89.91	-1.44	-0.42	-4.66	-2.91
**L59 × L77**	-98.74	-75.86	2.23	0.45	1.73	-1.25
**L59 × L75**	342.97	315.86	-2.45	-0.20	-0.30	3.95
**L59 × L80**	-253.07	-35.84	-0.03	-0.35	-5.90	-0.47
**P6 × L76**	490.48	531.74	-0.24	0.16	11.68	15.52
**P6 × L77**	596.97	585.00	1.19	1.03	18.90	18.74
**P6 × L75**	1262.43	1163.10	-0.56	0.39	25.91	27.55
**P6 × L80**	693.01	800.70	1.69	0.24	25.37	25.40
**L76 × L77**	-312.37	-286.80	-1.21	0.38	-10.36	-8.15
**L76 × L75**	299.76	354.87	1.40	-0.26	9.14	7.03
**L76 × L80**	-268.68	-386.46	-0.39	-0.42	-8.99	-11.76
**L77 × L75**	775.26	827.93	1.88	0.61	23.37	20.91
**L77 × L80**	284.96	249.58	0.02	0.45	7.61	8.27
**L75 × L80**	655.44	766.65	0.43	-0.19	17.93	20.04

GY: grain yield; PE: popping expansion; and PV: volume of expanded popcorn per hectare.

Considering the averages observed in the sites, the most outstanding hybrids for PE in the low-phosphorus environment were: P7 × L59, L59 × L77, P2 × L75, L77 × L75 and P6 × L80, and in the high-phosphorus environment: P6 × L77, P7 × L77, P2 × L77, P7 × P6 and L77 × L75 (Table 6). Such low agreement in the classification of hybrids between the different environments is explained by the significance of the SCA × E interaction (Table 4). In addition, according to Vencovsky and Barriga [16], a selection should be performed for each specific environment making it possible to select, under specific conditions, the best hybrids deemed efficient and responsive to phosphorus use.

Since PV is associated with GY and PE, the latter should present high average values, or at least one of them should be outstanding, with high average values. Thus, we may designate the hybrid combinations P6 × L75, P6 × L80, P7 × L80, P7 × L76, and L77 × L75 as having the most robust ŝ_ij_ estimates (Table 6). All of those combinations were included in the first group of the Scott-Knott algorithm, with the exception of L77 × L75, and all presented high average values for the volume of expanded popcorn per hectare, varying from 82.90 to 98,66 m^3^ha^-1^ in environments with low and high phosphorus, respectively.

Overall, the lines utilized in this diallel have genetic potential for the exploration of hybrids in terms of efficiency and responsiveness to soil phosphorus. Of the obtained hybrids, many were promising for individual characteristics. In conclusion, considering the predominance of genetic effects for the concomitantly analyzed characteristics, as well as their averages expressed under contrasting environments, the hybrid combinations that presented promising efficiency and responsiveness to phosphorus can be summarized to the P7 × L80, P7 × L59, P7 × L76, and P6 × L80 combinations.

## Conclusions

The quadratic components of GCA were superior under the high and low phosphorus environments in terms of popping expansion, demonstrating a predominance of additive effects to promote efficiency and responsiveness to phosphorus use. The quadratic components of SCA were superior under the high and low phosphorus environments in terms of grain yield and volume of expanded popcorn per hectare, demonstrating the predominance of non-additive effects for efficiency and responsiveness to phosphorus use. Thus, the best strategy to obtain genotypes that are efficient and responsive to phosphorus use involves the exploration of heterosis through the identification of superior popcorn hybrids, by using genitors that provide an accumulation of additive genes that promote popping expansion. Hybrids P7 × L80, P7 × L59, P7 × L76, and P6 × L80 were promising and should be evaluated as cultivation options in phosphorus-deficient soils.
